# These Graphene Experts Are Trying to Close the Reproducibility Gap in Two-Dimensional Materials Research

**DOI:** 10.1021/acscentsci.6c00777

**Published:** 2026-05-13

**Authors:** Mark Peplow

## Abstract

Too much work on graphene and related materials cannot be repeated, a problem that wastes time and holds back commercialization. New rules could help solve it.

## Graphical abstract

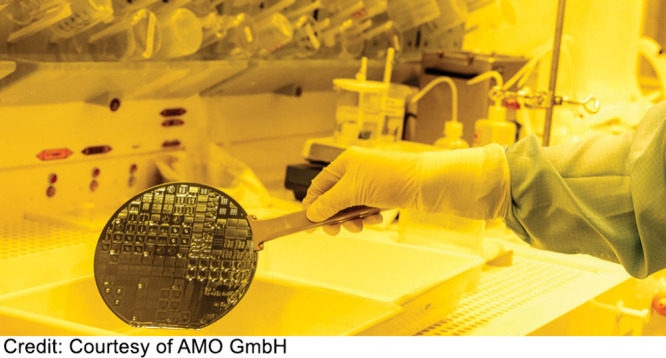

Ever since graphene’s debut in 2004, this atom-thin sheet of carbon has been touted as a revolutionary material because of its remarkable strength and electrical conductivity, as well as other outstanding properties. Its discovery triggered a wave of other 2D materials—including hexagonal boron nitride and molybdenum disulfide—many of which could serve as components in electronic devices.

**Figure:**
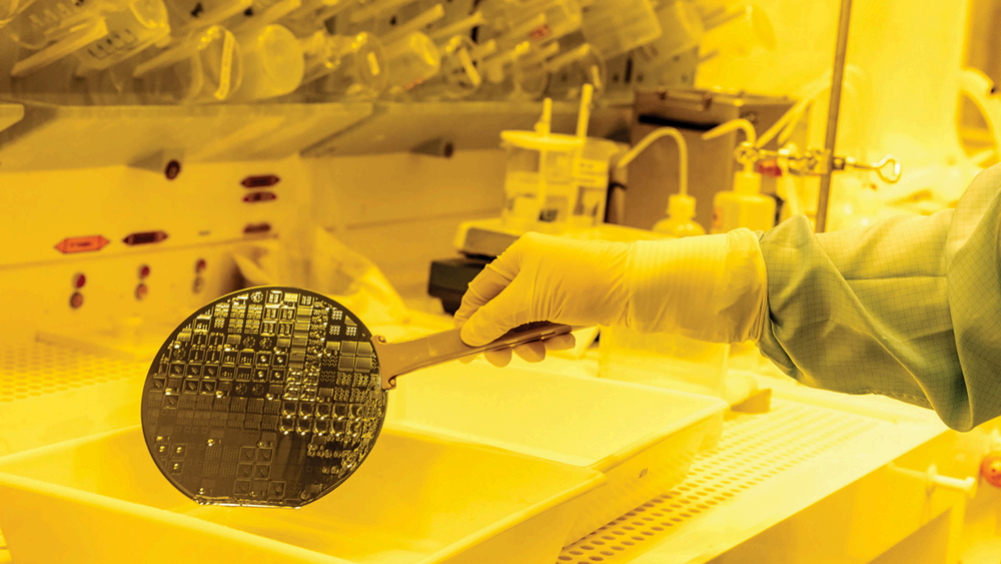
Companies incorporate graphene in electronic devices fabricated on wafers (shown above). Improving the reproducibility of graphene research could make this process easier. Credit: Courtesy of AMO GmbH.

But these materials can be difficult to work with; even minor variations in lab conditions can affect their properties. Researchers often find that results produced by another lab cannot be replicated in their own. Some believe that this “reproducibility gap” is slowing the translation of 2D materials into applications—a process known as technology transfer. “We can’t say we are working in a serious way on tech transfer if at the same time we’re not doing proper work on reporting and transparency,” says Peter Bøggild, who researches 2D materials at the Technical University of Denmark.

Bøggild brought together stakeholders from academia, industry, and funding bodies last year to develop practical guidelines aimed at closing this gap. This expert group proposed a template for researchers to record experimental methods in far more depth than is usually required for academic papers in order to capture the trials and tribulations of working with 2D materials.

Ediz Herkert, a postdoc researcher at the Institute of Photonic Sciences (ICFO) in Barcelona who was not involved in the group, thinks the guidelines could ultimately be a huge time-saver for the field. “It should feel like you have an experienced postdoc next to you who’s really explaining everything to you step by step,” he says.

Paying more attention to reproducibility could also stimulate technology transfer by making it easier for companies to adopt and scale up methods developed in academia. “These materials are complicated,” says Amaia Zurutuza, a coauthor of the recommendations. She is the scientific director at Graphenea, a firm based in San Sebastián, Spain, that manufactures graphene-based materials and chips. “If you don’t have the reproducibility part, then it becomes even more complicated.”

## Out in the open

All materials can suffer from contamination, but 2D materials are particularly susceptible because every single atom is exposed to the outside world. “The materials are literally from another dimension,” Bøggild says. “It’s inherently tricky to work with stuff that is open and cannot easily be protected from anything that lands on it.”

Subtle differences in preparation and handling methods can have a huge effect on 2D materials, and success may depend on tiny variations in temperature, humidity, or vibrations that are not always recorded in a paper’s methods.

When these materials are incorporated into devices such as transistors, researchers sometimes report only the very best “hero device,” ignoring dozens of failures that preceded it. Zurutuza says that is a big problem when industry tries to replicate the work. “So many times we find that it’s not as good as it seems,” she says. “But we don’t know if we did exactly the same procedure, because not all the information is there.”

To further complicate matters, many companies have used the word *graphene* as a catchall for a range of related materials. Some are single-layer sheets of carbon atoms, which are typically grown by chemical vapor deposition (CVD). Others, like graphene nanoplatelets, contain multiple layers and are cheaper to make but offer more modest properties.

**Figure:**
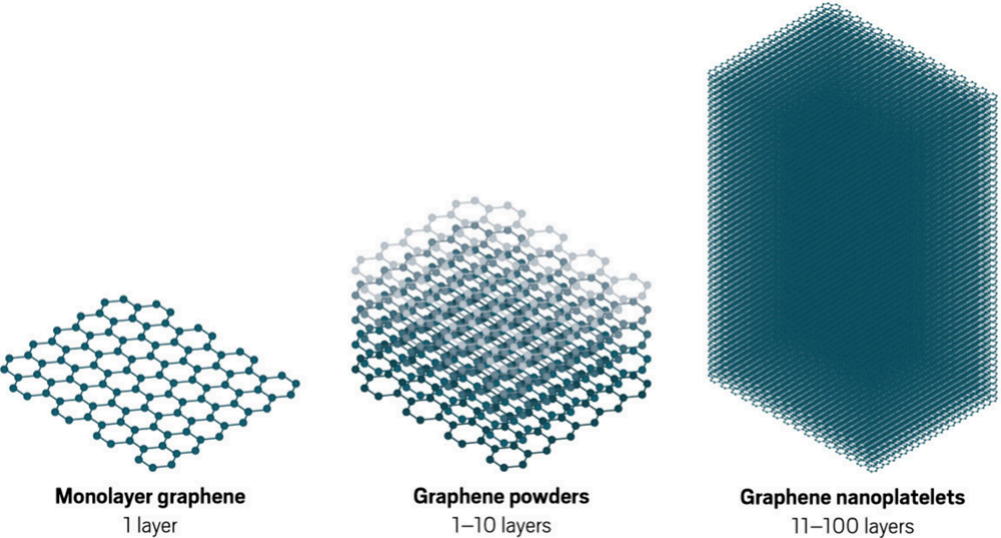
Researchers and companies often use the word *graphene* to refer to different materials—such as (from left) atom-thin monolayers, stacks of several layers, or nanoscale nuggets of carbon—that all have very different properties. Credit: Yang H. Ku/C&EN.

A 2018 study that assessed the quality of graphene sold by 60 commercial suppliers found wide variation in the size of the flakes, the number of layers, and purity. “When you buy graphene from commercial vendors, it’s a huge spread of materials, and it’s not high quality,” Bøggild says.

It can even be difficult to reliably measure graphene’s properties. Graphenea was involved in a study 4 years ago that took small graphene samples from the same CVD wafer and sent them to 17 laboratories for Raman spectroscopy analysis. Even this simple workhorse technique produced widely differing results that depended on the instrument, the measurement protocols, or lab conditions. “This field is huge, so systemic problems represent an enormous waste of resources—not just money, but also the time of postdocs and PhD students,” Bøggild says.

## A STEP at a time

The guidelines from Bøggild and other stakeholders outline a standardized template for experimental procedures (STEP), which is essentially a methods section on steroids. It guides researchers to break a procedure into a series of small steps and provide extensive details about things like materials, equipment, and variations in conditions such as pressure or temperature.

A research protocol that follows the template should offer troubleshooting guidance at each stage, list common problems that arose, and explain how the researchers dealt with them. It may even include photographs and videos to show particular procedures. The aim is to capture the tacit knowledge that researchers often share with lab colleagues but do not necessarily commit to paper. “You need to get all the dirt and the difficulties as part of the recipe,” Bøggild says.

“Everyone knows that your sample often doesn’t look as perfect as the one picture that you put into your paper,” Herkert says. “So I really like this greater honesty about reporting things.”

He and Jaime Díez Mérida, also a postdoc researcher at ICFO, hope to establish the STEP method as standard practice there and have already started adding their own procedures to a database shared with ICFO colleagues. They estimate that each one takes an entire day to produce. “It’s a lot of work, and maybe it’s hard initially to convince people to do it,” Herkert says. “But the time you invest saves a lot more time for other people. And if everyone else does it, it will save you time whenever you learn a new process.”

In fact, they argue that creating a STEP protocol has more-immediate benefits for the researchers who put it together because it forces them to focus on the most critical elements of the method and potentially reveals areas that can be improved.

In addition to STEP, Bøggild and his coauthors propose an initiative for making reproducibility goals more prominent in funding proposals and published papers. Known as the Reproducibility Charter (ReChart), it would act as a checklist that, for example, funders could use to allocate part of a grant for creating STEP protocols. Similarly, publishers could adopt ReChart to establish requirements for reproducibility reporting in a paper.

The ReChart approach is endorsed by Anders Smith, who coauthored the expert recommendations and is a funding manager at the Villum Foundation, Denmark’s second-largest private funder of scientific research. “We worry about whether it’s too difficult to get funding for reproducibility,” Smith says. “So we tell our grantees they are welcome to use part of their grant on such activities, and we actually have people creating very interesting new stuff by going back and looking at accepted results that maybe no one bothered to examine before.”

## The right direction

Some major graphene projects already have reproducibility goals baked into their work. For example, the European Union’s long-running Graphene Flagship program has funded the Belgium-based 2D Experimental Pilot Line and its successor project, the 2D-Pilot Line. A goal of these efforts is to develop wafer-scale fabrication processes that reliably incorporate high-quality graphene into electronic devices. That work could enable 2D materials to be adopted by the semiconductor industry. “So it’s moving in the right direction,” Zurutuza says.

**Figure:**
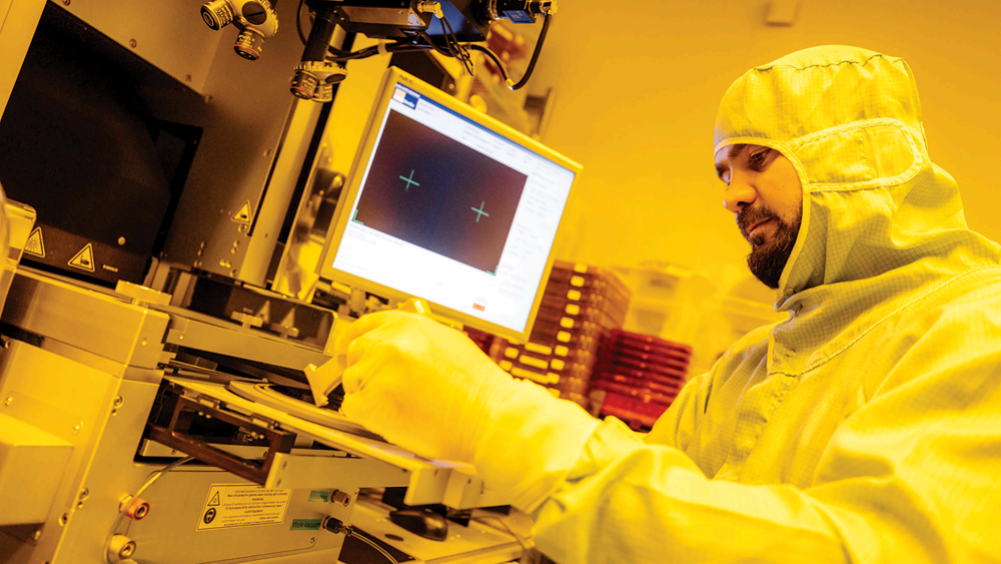
2D Experimental Pilot Line, funded by the European Union’s Graphene Flagship program, has developed reliable methods to deploy graphene in the semiconductor industry. Credit: Courtesy of AMO GmbH.

Bøggild says all the researchers, companies, funders, and publishers he has spoken to agree with the principles of the expert recommendations. The key challenge will be persuading enough of these 2D materials stakeholders to actually implement them. “Maybe it just needs a little push from me and 1,000 other people,” he says. “Even a small shift could matter a lot.”

Establishing that kind of rigorous focus on reproducibility could have a much wider impact, Herkert says. “The strength of this protocol is that it’s really not just limited to the field of 2D materials,” he says. “It’s a template that can be useful in many different fields, basically everything that is related to nanofabrication and clean room work.”

*Mark Peplow is a freelance contributor to* Chemical & Engineering News, *an independent news publication of the American Chemical Society.*

